# Medical code prediction via capsule networks and ICD knowledge

**DOI:** 10.1186/s12911-021-01426-9

**Published:** 2021-07-30

**Authors:** Weidong Bao, Hongfei Lin, Yijia Zhang, Jian Wang, Shaowu Zhang

**Affiliations:** 1 School of Information Engineering, Dalian Ocean University, Dalian, China; 2 College of Computer Science and Technology, Dalian University of Technology, Dalian, China

**Keywords:** Clinical notes, Medical code prediction, Capsule network, Domain knowledge

## Abstract

**Background:**

Clinical notes record the health status, clinical manifestations and other detailed information of each patient. The International Classification of Diseases (ICD) codes are important labels for electronic health records. Automatic medical codes assignment to clinical notes through the deep learning model can not only improve work efficiency and accelerate the development of medical informatization but also facilitate the resolution of many issues related to medical insurance. Recently, neural network-based methods have been proposed for the automatic medical code assignment. However, in the medical field, clinical notes are usually long documents and contain many complex sentences, most of the current methods cannot effective in learning the representation of potential features from document text.

**Methods:**

In this paper, we propose a hybrid capsule network model. Specifically, we use bi-directional LSTM (Bi-LSTM) with forwarding and backward directions to merge the information from both sides of the sequence. The label embedding framework embeds the text and labels together to leverage the label information. We then use a dynamic routing algorithm in the capsule network to extract valuable features for medical code prediction task.

**Results:**

We applied our model to the task of automatic medical codes assignment to clinical notes and conducted a series of experiments based on MIMIC-III data. The experimental results show that our method achieves a micro F1-score of 67.5% on MIMIC-III dataset, which outperforms the other state-of-the-art methods.

**Conclusions:**

The proposed model employed the dynamic routing algorithm and label embedding framework can effectively capture the important features across sentences. Both Capsule networks and domain knowledge are helpful for medical code prediction task.

## Background

Clinical notes are written by the professional medical staff and record various information of patients in the hospital such as their medical history, lifestyle, symptoms, treatment, and medications. Clinical notes facilitate quick access to the patients’ information by the medical staff and significantly contribute to the treatment process of the patients [1]. A medical code refers to a process of converting the clinical notes from the medical ontology into a group of medical codes. The most popular medical codes are the international disease codes ICD 9 and ICD 10. The ICD codes standardize and format the disease names which is the basis for applying clinical information systems such as medical information and hospital information management [2]. The ICD code enables the most comprehensive sharing of disease information, reflecting the nation's health status. It is also very helpful for medical research and teaching. The ICD is the management basis of hospital medical treatments and administration. Disease classification is also one of the vital basis of medical expenses control which is conducive to cost management and insurance reimbursements. The international disease code ICD 9 approximately includes 138,000 codes.

The process of assigning the medical codes to the clinical notes is often performed by trained professionals. Clinical notes, however, contain complex medical terminology and often include spelling errors and abbreviations. Therefore, reading the notes and assigning the corresponding codes is usually challenging for the professionals and requires a significant amount of time, human and financial resources. An automatic medical code assignment system can significantly improve this process and reduce human errors in ICD code assignment [3].

There exist several methods for automatic medical codes assignment to clinical notes. These methods are categorized into rule-based methods, machine learning methods, and neural network methods. Instances are K-nearest neighbours, correlation feedback, support vector machines [4], Bayesian ridge regression [5], and various neural network models [6, 7]. In [8], the author developed a method based on the hierarchical tree structure of the ICD-9 ontology. Larkey et al. [9] also used classifiers to obtain the candidate ICD labels, but the classifiers need to adjust all the parameters artificially. Koopman et al. [10] used SVM for automatic ICD-10 classification of cancer. Perotte et al. [11] mention a hierarchy based SVM model to predict the ICD codes for the discharge summaries. Lita et al. Further in [5] the ICD code classification performance of the SVM was investigated. In general, machine learning methods are time-consuming to design the specific features, that are also significantly dependent on professional competence and human resources.

With the development of artificial intelligence, deep learning methods have demonstrated incredible potential for automatic medical codes assignment and achieving a higher level of performance in the NLP tasks [12]. Neural networks with attention mechanisms [13] have achieved high performance in multi-label classification tasks [14]. Attention mechanisms [15] use one or more layers of the neurons to identify specific words and assign them different weights to obtain more accurate classification results. However, these methods are not effective at dealing with long texts in document classification tasks [16]. In general, the document text is much longer than sentence text, especially as some document texts contain many complex sentences. How to learn potential feature representations from document text is still considered as an open problem in the NLP domain.

Using label embedding has proven effective in various domains and tasks such as image classification in computer vision [17], multi-modal learning between images and text [18], and text recognition in images [19]. Label embedding also shows excellent performance in zero-shot learning tasks [20], where certain classes are not visible and capturing label correlation in the embedding space can improve predictive performance. Label embedding for the text multi-label classification has been studied in the heterogeneous networks [21]. Word embedding is an efficient intermediate representation for capturing semantic rules between the words for learning text sequence representations. In 2017, Hinton proposed the capsule network architecture [22], modifying the traditional CNN that can be used for performance improvement of the ICD codes assignment. Therefore, the label embedding framework and capsule network are both useful for automatic medical codes assignment tasks.

In this paper, we propose a hybrid model based on the capsule network and label embedding framework to predict the ICD codes from the clinical notes. The model effectively captures the important syntactic features between the sentences and learns a comprehensive representation of the potential features throughout the document text. Our proposed model uses a dynamic routing algorithm to capture more specific features in the clinical notes to improve accuracy, instead of filtering the features using a pooling method that may lose important information. A label embedding framework is also proposed to incorporate more information from the labels. Our approach is evaluated on the MIMIC-III public corpus and the experimental results confirm that the model achieves state-of-the-art performance.

The rest of the paper is organized as follows. We briefly describe the medical code assignment task followed by the details of our proposed model in method section. Then in results and discussions section, we show the experimental results and discussion of the MIMIC-III corpus. Finally, in conclusions section we summarize our work and conclude.

## Method

### Medical codes assignment

The automatic medical codes assignment is a long-term and challenging area of research. In recent years, many researchers explore the use of textual data for automatic ICD codes assignment [23], intended to automatically and accurately predict the corresponding medical code from the clinical notes. In this paper, we choose the MIMIC-III corpus for our experimental study.

**Fig. 1:**
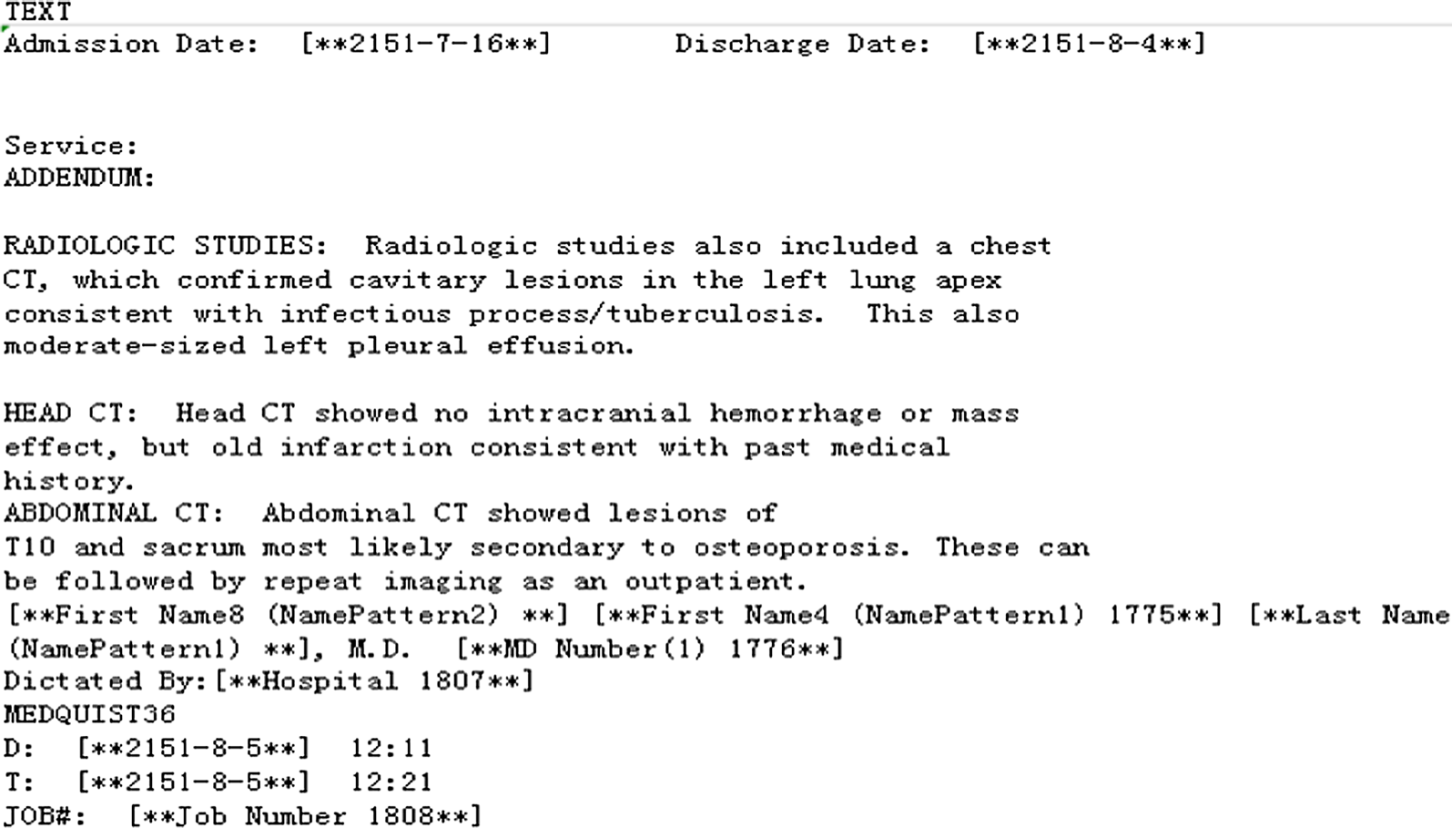
Note events in MIMIC-III corpus

The Medical Information Mart for Intensive Care (MIMIC-III) is a large, single-centre database comprising information relating to the patients admitted to critical care units at a large tertiary care hospital [2]. Figure 1 and Table 1 provide examples of the MIMIC-III corpus. Note events contain the patient's clinical notes. DIAGNOSE_ICD contains the ICD codes corresponding to the patient's clinical notes. MIMIC-III contains 59,652 discharge records from the medical centre. There are 942 unique 3-digit ICD-9 codes in the dataset. However, the distribution of the codes is highly unbalanced. Common codes account for 26% of all codes and rare codes only account for 1%. On average, each MIMIC-III discharge note contains about 1500 words.

**Table 1:** DIAGNOSES_ICD in MIMIC-III corpus

ROW_ID	SUBJECT_ID	HADM_ID	SEQ_NUM	ICD9_CODE
1297	109	172,335	1	40,301
1298	109	172,335	2	486
1299	109	172,335	3	58,281
1300	109	172,335	4	5855
1301	109	172,335	5	4254
1302	109	172,335	6	2762
1303	109	172,335	7	45,829
1304	109	172,335	8	2875

Our dataset is a collection of clinical notes. Each clinical note X consists of many words, and each X has a set of medical codes M corresponding to that note. Besides, there is an external knowledge source L (Table 2), which contains a basic description of each ICD code. Our goal is to add the label embedding framework to the hybrid model to predict the relevant medical codes M. The entire experiment is a multi-label text classification task.

**Table 2:** Example of 3-digit ICD9 description

ICD code	Description
001	Cholera
002	Typhoid and paratyphoid fevers
003	Other salmonella inflections
004	Shigellosis
005	Other food poisoning (bacterial)
006	Amebiasis
007	Other protozoal intestinal diseases

### Our model

**Fig. 2:**
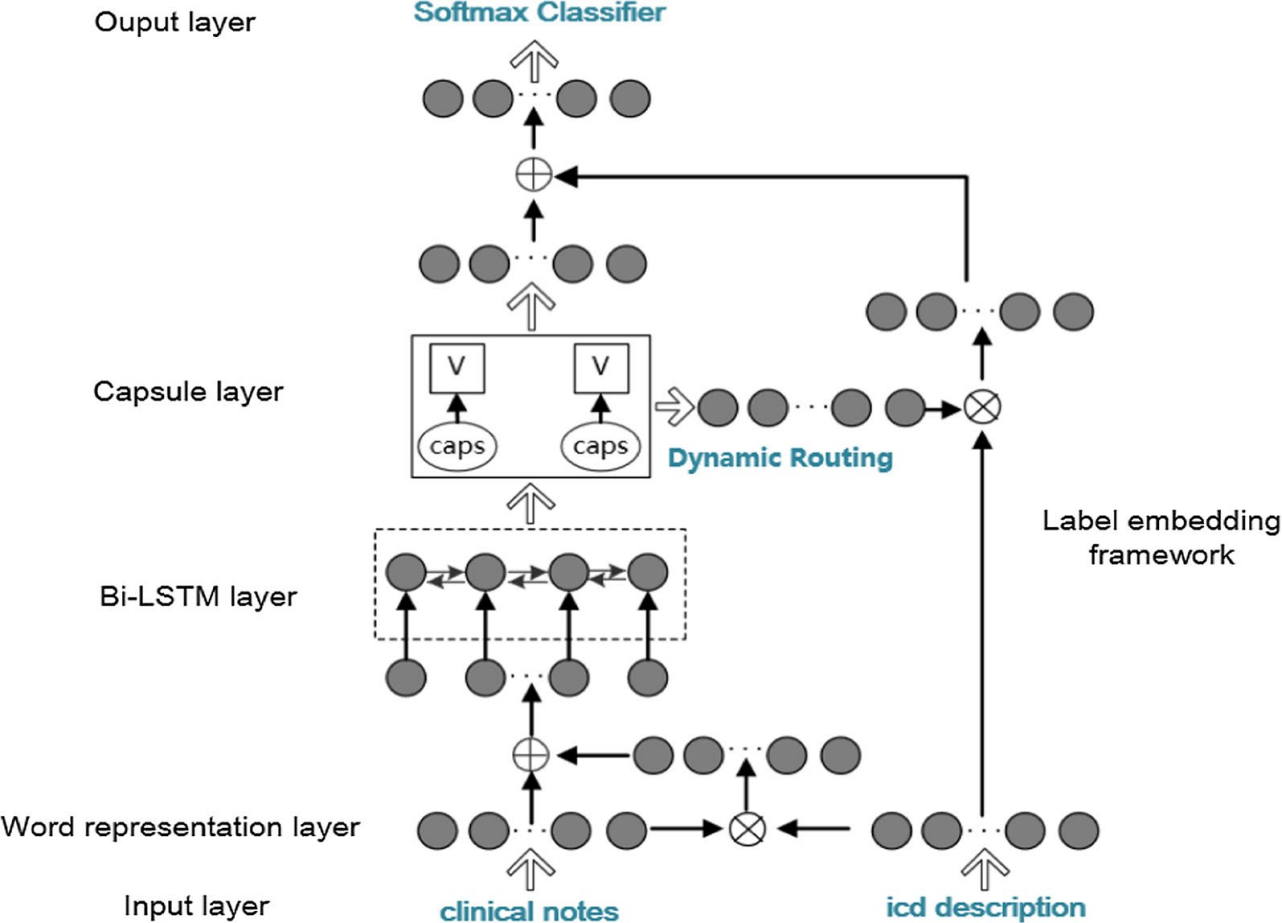
Schematic overview of our model

A schematic overview of our model architecture is shown in Fig. 2. Our model consists of three main parts, including the Bi-LSTM [24] layer, the label embedding framework, and the capsule network (CapsNet) layer.

The inputs of our model are text sequences. The word embedding layer generates a distributed representation vector containing semantic information for each word. We use bi-directional LSTM (Bi-LSTM) with forwarding and backward directions to merge the information from both sides of the sequence. It can capture the contextual information of each word to obtain a comprehensive sequence of the output vectors. The label embedding framework embeds the text and labels together to leverage the label information. We then use a dynamic routing algorithm in the capsule network to extract more valuable features. Finally, we use a fully connected layer and SoftMax function to implement the text classification. The details of our model are presented in the following sections.

#### Word representations

The distributed representation method, also known as word embedding [25, 26], is based on the premise that semantically similar words have similar semantics. Word embedding has been widely used in the BioNLP domain to effectively capture the underlying semantic information of the words. Since the clinical notes are usually written by the medical professionals, we adopt the distributed representation method to obtain a word vector that approximates the objective word meaning more closely. In this paper, the pre-trained word embeddings are downloaded from nlplab, using the word2vec tool to train the word vectors from PubMed and PMC texts.

#### Bi-LSTM

In a sentence, the semantics of the preceding and following words are related. Recurrent Neural Network (RNN) is one of the neural network models [27] which aims to capture the sequential patterns presented in data. It has however limited due to the gradient vanishing problem. The LSTM is a variant of RNN [28], which addresses the issue of long-term dependencies by introducing the gate mechanism and the memory cell. It can keep the long dependent information and conquer the gradient vanishing issue. The LSTM model overcomes the vanishing gradient problem by introducing gating mechanisms. Therefore, it is suitable to capture the long-term dependency feature.

The LSTM unit consists of three components: the input gate ${\mathrm{i}}_{\mathrm{t}}$, the forget gate ${\mathrm{f}}_{\mathrm{t}}$ and the output gate ${o}_{t}$. At the time step $\mathrm{t}$, the LSTM unit utilizes the input word ${\mathrm{x}}_{\mathrm{t}}$, the previous hidden state ${\mathrm{h}}_{\mathrm{t}-1}$ and the previous cell state ${\mathrm{c}}_{\mathrm{t}-1}$ to calculate the current hidden state ${\mathrm{h}}_{\mathrm{t}}$ and cell state ${c}_{t}$ [24]. The equations are as follows:

1 $${f}_{t}=\sigma \left({W}_{f}{x}_{t}+{U}_{f}{h}_{t-1}+{b}_{f}\right)$$

2 $${g}_{t}=\mathrm{tanh}\left({W}_{g}{x}_{t}+{U}_{g}{h}_{t-1}+{b}_{g}\right)$$

3 $${o}_{t}=\sigma \left({W}_{o}{x}_{t}+{U}_{o}{h}_{t-1}+{b}_{o}\right)$$

4 $${i}_{t}=\sigma \left({W}_{i}{x}_{t}+{U}_{i}{h}_{t-1}+{b}_{i}\right)$$

5 $${c}_{t}={f}_{t}\odot {c}_{t-1}+{i}_{t}\odot {g}_{t}$$

6 $${h}_{t}={o}_{t}\odot \mathrm{tanh}\left({c}_{t}\right)$$

where W, U is weight, b is bias term, $\odot$ denote element-wise multiplication.

**Fig. 3:**
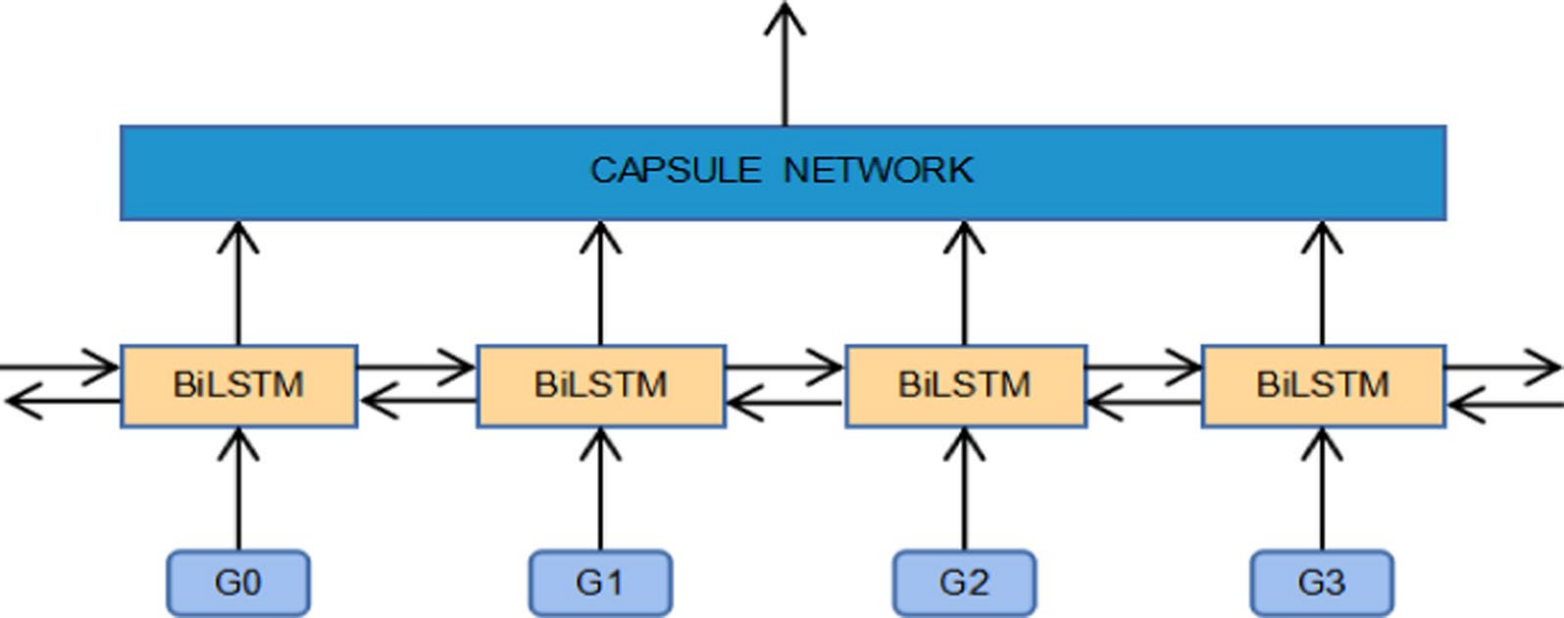
The architecture of CapsNet with Bi-LSTM

Figure 3 shows the architecture of the CapsNet hybrid model. In this paper, we use Bi-directional LSTM (Bi-LSTM) to integrate the information from both sides of the sequence in a forward and backward manner. It takes the word representation sequence of the text as the input and then outputs a new word representation sequence that captures the contextual information of each word in the text.

#### Label embedding framework

In this section, we use $\circ$ for function composition, $S={\left\{\left({X}_{n},{Y}_{n}\right)\right\}}_{n=1}^{N}$ represents a training set, ${X}_{\mathrm{i}}$ represents the sequence of the clinical notes and ${Y}_{i}$ is the corresponding ICD codes. The objective of the label embedding framework hybrid model is to learn a mapping from X to Y that makes the formula $min\frac{1}{N}\sum_{n=1}^{N} \delta \left({Y}_{n},f\left({X}_{n}\right)\right)$ minimal, where $\delta$ is the loss function that measures the loss between $f\left({X}_{\mathrm{i}}\right)$ and ${Y}_{i}$.

**Fig. 4:**
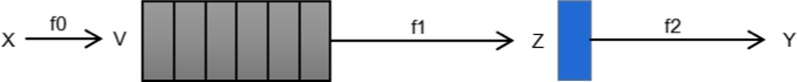
Common methods to text classification

Text classification is seen as a combination of three functions $f={f}_{0}^{\circ }{f}_{1}^{\circ }{f}_{2}$. Figure 4 shows the common methods of text classification, where ${f}_{0}$ is a word embedding function, ${f}_{1}$ is a function to aggregate word embedding to get text representation, and ${f}_{2}$ is a function for classifying using text representation. There are two broad categories of approaches to designing ${f}_{1}$. One is to treat the process as a “black box” that learns mappings using various deep learning models [29, 30], while the other approaches use simple max pooling or mean pooling method. As it is seen, only ${f}_{2}$ used the label information, hence the impact of the label is indirect for both ${f}_{0}$ and ${f}_{1}$. Here, we propose adding the label information in each process.

**Fig. 5:**
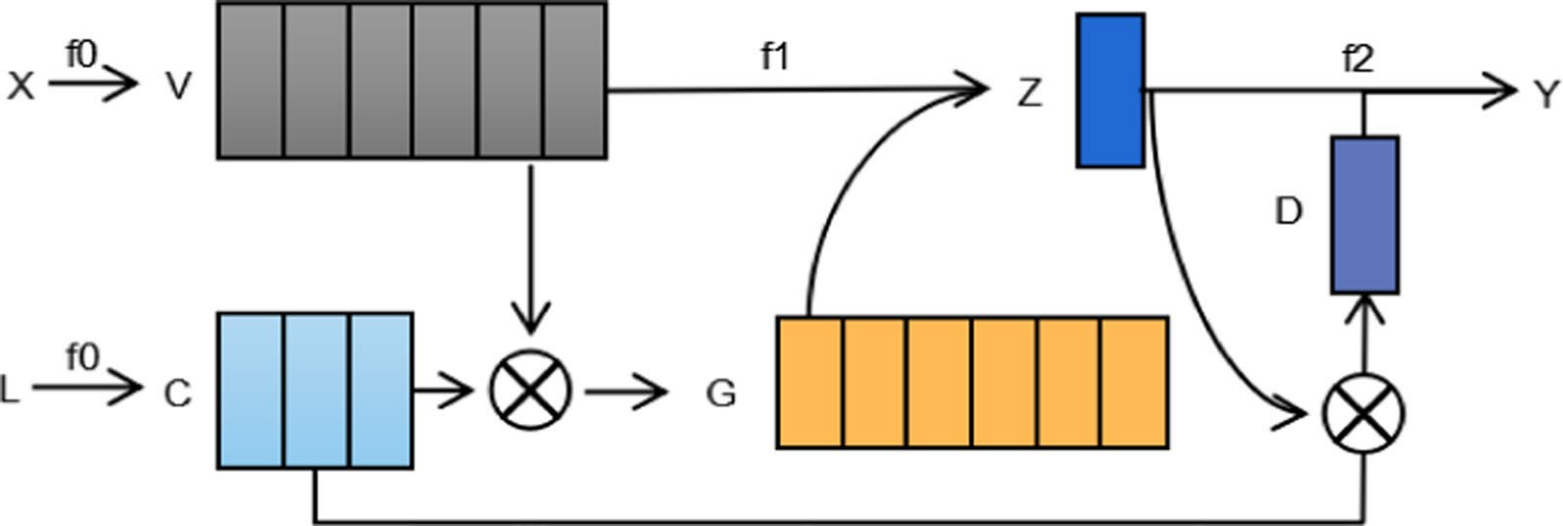
Joint label embedding method

Figure 5 shows how we joint label embedding framework. In ${f}_{0}$, the model learns the embedding of labels as the key point to influence the word embedding. In ${f}_{1}$, the model uses the correlation between the labels and the words for word embedding splicing. Specifically, ${C}_{i}$ represents the $i\text{ -th}$ category of label embedding as the matrix of label embedding. We embed both words and labels into a joint space. The calculation equations are shown as follows:

7 $$G=C\otimes V$$

8 $$Z={f}_{1}\left(G\oplus V\right)$$

9 $$D=Z\otimes C$$

10 $$Y={f}_{2}(Z\oplus D)$$

where $\otimes$ represents cosine similarity between vectors, $\oplus$ denotes concatenate among $G$ and $V$, $Z$ and $D$. In the above $C$ and $V$ similarities are concatenated to word embedding and fed into Bi-LSTM. The capsule network is then used to extract the features and output the [N_CAP, CAP_DIM] features, the EMB_DIM encoded sentences are combined with the label embedding to calculate the cosine similarity. The similarity is then added to the classification results.

#### Capsule network

**Fig. 6:**
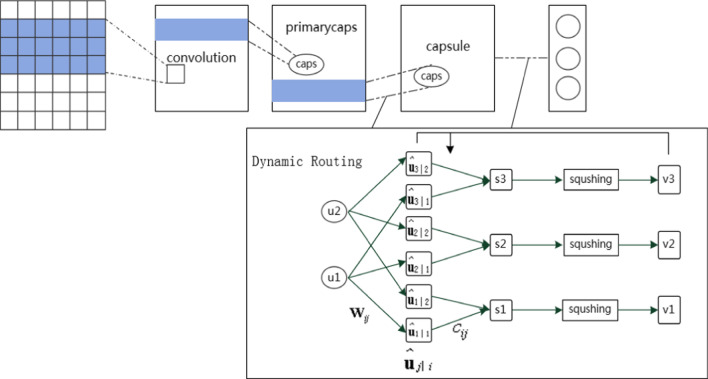
capsule network model architecture

In deep learning models, spatial patterns are aggregated at a lower level which helps to represent higher-level concepts. For example, CNN builds a convolutional feature detector to extract patterns from the local sequence windows and uses max pooling to select the features. The CNN extracts feature patterns at different levels in a hierarchical manner. However, since the convolution operator in the CNN is a weighted representation of the lower layers, it is difficult to represent the features of a complex object where it enters the upper layer. In this work, capsule networks are employed to learn the potential feature representation. Our capsule network architecture is shown in Fig. 6.

In the convolution layer of the capsule network, extracting N-gram features at different positions of a sentence through various convolutional filters. The N-gram size ${\mathrm{K}}_{1}$ slide on the sentence to detect the features at different positions. The filter of the convolution operation ${W}^{a}\in {R}^{{K}_{1}\times e}$ is then convolved with the word window ${\mathbf{X}}_{i}$ at every position to generate a column feature map ${\mathbf{m}}^{a}$, ${m}_{i}^{a}\in R$ and $\mathbf{M}$ can be calculated as follows:

11 $${m}_{i}^{a}=f\left({X}_{i}^{ \circ }{W}^{a}+{\mathbf{b}}_{0}\right)$$

12 $$\mathbf{M}=\left[{\mathbf{m}}_{1},{\mathbf{m}}_{2},\dots ,{\mathbf{m}}_{\mathrm{N}}\right]$$

where $\circ$ denotes element-wise multiplication, ${\mathbf{b}}_{0}$ is a bias term, and $f$ is a nonlinear activate function.

At the primary capsule layer, the capsule replaces the CNNs' scalar output feature detector with a vector-output capsule, it can retain more characteristic information such as the local order of words and the semantic representation of words. Let ${p}_{i}\in {R}^{d}$ denote the capsule parameter information, $d$ be the dimension of the capsule, ${W}^{b}\in {R}^{N\times d}$ be the filter shared among different sliding windows. A window slides over each N-gram vector for each matrix multiplication, denoted as ${\mathbf{M}}_{i}\in {R}^{N}$. The filter ${W}^{b}$ multiplies each N-gram vector in ${\mathbf{M}}_{i}$ to produce a column-list of the capsules $\mathbf{p}$. Each capsule ${p}_{i}\in {R}^{d}$ and $\mathbf{p}$ are calculated as follows:

13 $${p}_{i}=g\left({W}^{b}{\mathbf{M}}_{i}+{\mathbf{b}}_{1}\right)$$

14 $$\mathbf{P}=\left[{\mathbf{p}}_{1},{\mathbf{p}}_{2},\dots ,{\mathbf{p}}_{\mathrm{C}}\right]$$

where $g$ is a nonlinear squash function, and ${\mathbf{b}}_{1}$ is a bias term.

The capsule network uses a nonlinear function called "squashing". This nonlinear function ensures that the length of the short vector can be shortened to almost zero, while the length of the long vector is compressed to close to but no more than 1. The following is the expression for this nonlinear function:

15 $${\mathbf v}_j=\frac{\left\|{\mathbf s}_j\right\|^2}{1+\left\|{\mathbf s}_j\right\|^2}\frac{{\mathbf s}_j}{\left\|{\mathbf s}_j\right\|}$$

where ${\mathbf{v}}_{j}$ is the vector output of capsule $\mathrm{j}$, and ${\mathbf{s}}_{j}$ is the input.

The total input for all capsules except the first layer capsule is a weighted sum of all vectors from the below layer of capsules generated by multiplying the output of the capsule layer by a weight matrix, as follows:

16 $${\mathbf{s}}_{j}=\sum_{i} {c}_{ij}{\widehat{\mathbf{u}}}_{j\mid i}$$

17 $${\widehat{\mathbf{u}}}_{j\mid i}={\mathbf{W}}_{ij}{\mathbf{u}}_{i}$$

where ${c}_{ij}$ is the coupling coefficient and is iteratively updated and determined by the dynamic routing process. The calculation method of ${c}_{ij}$ is shown as follows:

18 $${c}_{ij}=\frac{\mathrm{exp}\left({b}_{ij}\right)}{\sum_{k} \mathrm{exp}\left({b}_{ik}\right)}$$

19 $${b}_{ij}\leftarrow {b}_{ij}+{\widehat{\mathbf{u}}}_{j\mid i}\cdot {\mathbf{v}}_{j}$$

The sum of the coupling coefficients between all capsules is 1 and ${b}_{ij}$ is initialized to 0, where $i\in [1,a]$, $j\in [1,k]$, and $k$ is the number of classes.

## Results and discussions

### Datasets and evaluation metrics

We choose MIMIC-III dataset to evaluate our model. The total number of discharge summaries is 52,722, with an average of 1500 words per discharge summary. We preprocessed the data to remove the discontinued words, punctuation marks, and low-frequency words. The top three digits of the ICD codes for all patient visits were also aggregated then selected 344 codes from them. ICD 9 codes descriptions contain the ICD codes and the corresponding disease name descriptions. For 344 ICD codes, we found their corresponding descriptions and we used Micro F1, Macro F1, Test Loss, and Top-10 Recall to measure the performance of the model for the automatic code assignment tasks on the MIMIC-III dataset. Top-10 Recall is defined as the number of correct medical codes ranked in the top max(10, **|**M**|**) of the predicted results divided by **|**M**|**, where **|**M**|** is the number of medical codes for the note, and averaged over all test notes. The F1 score is the harmonic mean between the precision and the recall, in Micro-averaging, each forecast has the same weight and calculates the average metric over all instances. Therefore, the final result is dominated by medical codes with higher frequencies. Micro F1 gives a good overall indication of the model's performance. The macro-average metric calculates the value for each medical code separately and then takes the average of all codes. Since all the labels are given equal weights, the macro-average metric places emphasis on the prediction of the rare medical codes. However, the distribution of medical codes in MIMIC-III is not even, with the most common 10 codes occurring 26% of all codes and the least common 437 codes occurring only 1% of all codes. Therefore, the Macro F1 value in the experimental results is much lower than that of the Micro F1 value.

### Experimental settings

In our experiments, our model is implemented by keras with TensorFlow backend. To accommodate the computational complexity, we initially set the model dimension in word embedding to 200, the Bidirectional LSTM hidden units to 300, and the batch size to 16. In the capsule network, the capsule dimension is 50, the dynamic routing iteration number is 3, and the learning rate is set 0.01. All parameters of the model are optimized using Adam [31] to minimize the loss of categorical cross-entropy. To ensure that other experimental results can be reproduced, we perform experiments using the same dataset and the same splitting method. In the previous study [32], the dataset was divided into a training set, a validation set, and a test set of 0.7, 0.1, and 0.2, respectively. In our experiment, we keep the same splitting of dataset and compared with the previous studies. An early stop mechanism is also used to decide where to stop the training.

### Experimental results

#### Baseline methods

We selected three baseline models for comparison including CNN, LSTM [27], and capsule network. The model uses word embedding trained by the Word2Vec tool as the input. Here we briefly describe the selected baseline models:

Convolutional Neural Network (CNN): CNN has been successfully applied in NLP to extract the features which is a traditional method for text classification [33]. CNN is composed of an input layer, convolutional layer, activation function, pooling layer, and fully connected layer. In our work, we use three convolutional layers, the kernel size is 3, 4, and 5, respectively. Hidden dim is set to 200 and the dropout probability is set to 0.2.

Long Short-Term Memory (LSTM): LSTM is a special RNN which is mainly used to solve the problem of gradient vanishing and gradient explosion in the training process of the long sequences. Compared with the general CNN, LSTM performs better on the longer sequences. An LSTM unit has three gates including forget gate, input gate, and output gate. In our experiments, we set the hidden dim to 200, the batch size is set to 32, and the dropout is set to 0.2.

Capsule Network (CapsNet): CapsNet was originally used in the imaging field and showed excellent performance. In our work, we use a CNN-based capsule network to process text, where the scalar output of the traditional neural network is replaced with the vector output of the capsule network, and dynamic routing algorithm is used instead of the traditional pooling layer to train the neural network. The capsule vector dimension is set to 50 and the dynamic routing iteration is set to 3. The capsule network mainly consists of the convolutional layer, primary capsule layer, capsule layer and fully connected capsule layer.

We compared our proposed BiCapsNetLE method with CNN, LSTM, and capsule network models [11]. The performance of each model on the MIMIC-III dataset is shown in Table 3. The performance of the CNN model in the data set is general, with a Micro F1 score of 62.6%, the capsule network effectively improves the Micro F1 score from 62.6 to 64.7%. The results show that the capsule network's dynamic routing algorithm can capture more features from the textual information. The LSTM model relies on three gating units to have much better results for processing long text, with a Micro F1 value of 65.3%. Our proposed model is superior to the other three models in terms of all micro-measurement indicators. The value of Micro F1 is increased by 4.9% compared to CNN, 2.2% compared to LSTM, and 2.8% compared to capsule network. Macro F1 is 4.7% higher than CapsNet, 5.1% higher than CNN, 6.1% higher than LSTM. The result indicates that our model has a better improvement on rare coding prediction, and it achieves the smallest loss value among all models. This means the misclassification rate of our model is the lowest.

**Table 3:** The performance of our model and the baseline models on the MIMIC-III dataset

Methods	Macro F1 (%)	Micro F1 (%)	Test loss (%)	Top-10 recall (%)
CNN	21.4	62.6	4.0	75.3
LSTM	20.4	65.3	3.2	77.2
CapsNet	21.8	64.7	3.5	76.1
BiCapsNetLE	26.5	67.5	2.9	82.3

**Fig. 7:**
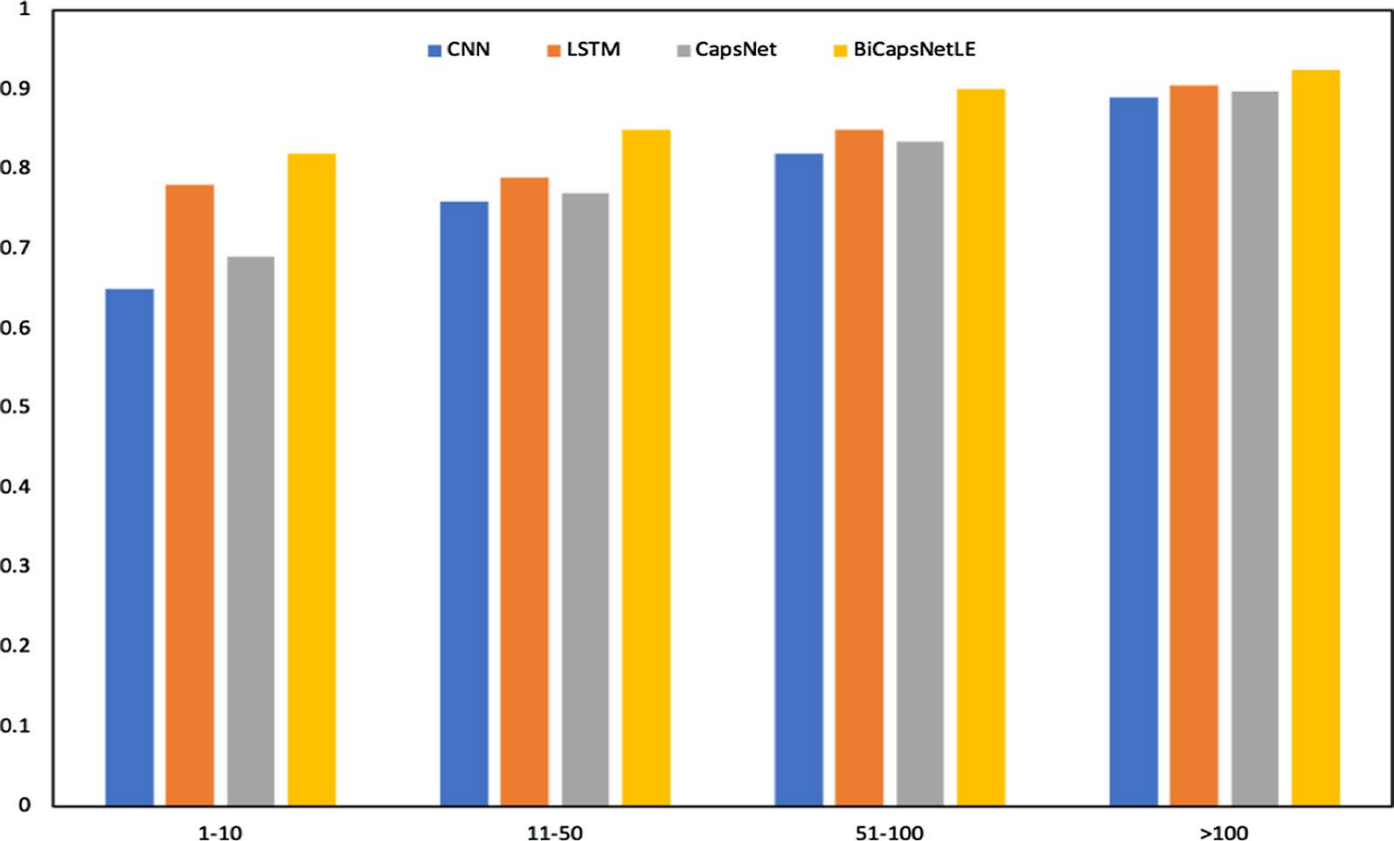
Macro AUC by label frequency groups for different models. X-axis represents the label frequency groups and y-axis represents the Macro AUC

To visually demonstrate the impact of introducing the external knowledge sources on the experimental results, we divided medical codes into four groups according to the frequency of their occurrence in the dataset, i.e., [1, 10], [11,50], [51,100], and [100, + ∞). We further calculate the Macro AUC for all models on each group, where the Macro AUC is the unweighted average AUC over all labels. The results in Fig. 7 indicate that our model can improve the AUC of low-frequency labels in the dataset. In particular, since the medical codes with higher frequency have more examples in the learning process, they benefit less from the external sources. Conversely, because the rare codes have few examples, they can learn more from the external knowledge sources.

#### Comparison with related work

We compared our model with several state-of-the-art medical code assignment methods. The results are shown in Table 4. In [32], Bai et al. presented a model that incorporates the external resources of Wikipedia knowledge framework (KSI) into the model to predict ICD codes and implements the optimal performance on the MIMIC dataset. In [16], Mullenbach et al. presented the Convolutional Attention for Multi-Label classification (CAML) model which combines the attention mechanism with CNN to achieve state-of-the-art performance in medical codes prediction. In [13], Wang et al. presented a label embedding attentive model (LEAM) to improve the text classification.

**Table 4:** Comparison with related works

Methods	Macro F1 (%)	Micro F1 (%)	Test loss (%)	Top-10 recall (%)
LRKSI [32]	19.6	55.7	5.5	73.8
RNNKSI [32]	24.4	66.2	3.0	79.8
CNNKSI [32]	23.7	63.7	3.9	77.5
CAML [16]	25.7	65.6	3.2	80.6
LEAM [13]	24.9	64.9	–	–
BiCapsNetLE	26.5	67.5	2.9	82.3

Compared to other deep neural network-based approaches, our method achieves the highest Micro F1 and Macro F1 scores of 67.5% and 26.5% for the MIMIC-III dataset. Our proposed model also outperforms other competitive models in all measurements. Comparing the results from other experiments, we improved Micro F1 by 11.8% compared to LRKSI, 1.3% compared to RNNKSI, 3.8% compared to CNNKSI, 1.9% compared to CAML, 2.6% compared to LEAM, and Macro F1 is increased by 6.9% compared to LRKSI, 2.1% compared to RNNKSI, 2.8% compared to CNNKSI, 0.8% compared to CAML, 1.6% compared to LEAM. Our proposed model also achieved the smallest loss value amongst the investigated models. In addition to using label embedding framework to acquire more important features, we use the Bi-LSTM and capsule network to automatically and efficiently capture the valuable features from documents. More importantly, our model is relatively simple, unlike the integrated model which requires the construction of a large number of neural network models that requires more time and effort to be integrated.

#### Ablation study

To examine the contribution of each component of the model, we conducted an ablation study. The experimental results are shown in Table 5.

**Table 5:** Ablation studies for our model

Methods	Macro F1 (%)	Micro F1 (%)	Test loss (%)	Top-10 recall (%)
CapsNet	21.8	64.7	3.5	76.1
BiCapsNet	23.1	67.0	3.1	81.9
BiCapsNetLE	26.5	67.5	2.9	82.3

It is seen that removing the Bi-LSTM layer or the label embedding framework results in reducing the model's performance. This suggests that both layers can learn effective features. By adding the Bi-LSTM layer to the capsule network model, the Micro F1 value is increased by 2.3%, and by adding the label embedding framework, the Micro F1 is increased by another 0.5%.

Using the capsule network alone also leads to the smallest F1 score and the largest loss. This is mainly due to its intrinsic shortcomings in dealing with long texts. In the MIMIC-III dataset, the average length of the discharge summaries exceeds 1000 words. The capsule network cannot remember features of long text sequences well. This may lead to poor performance. In particular, it is seen that when the capsule network is combined with the Bi-LSTM layer, the performance has been greatly improved. This indicates the effectiveness of Bi-LSTM and shows that the hybrid model can learn more long text features. The label embedding framework can significantly improve the scores of other test results, the results illustrate that the label embedding framework can provide more useful label information to add to the model label prediction task, and also effectively improves the sparsity of rare ICD codes distribution.

#### Relational visualization

To show the strength of the connection between the capsule layers, we use the primary capsule layer to directly connect the last fully connected capsule layer, where the primary capsule represents the N-gram words in the form of a capsule. The connection strength can show the importance of each primary capsule for the text category, like attention mechanism. This allows the capsule network to recognize multiple medical codes in the clinical notes.

In Table 6, we have manually annotated several words related to the ICD disease labels (e.g., "acid" and "atrium"), highlighted in bold for reference. We used WordCloud to visualize 3-g words for the ICD 276 and ICD 427 categories. The stronger the connection strength, the larger the font size. In Table 7, from the results, we observe that the capsule network correctly identifies and clusters the important words about the text category. Histograms are also used to show the strength of the connections between the primary capsule and the fully connected capsule.

**Table 6:** Partial clinical note

Clinical note	
**Fluids**, **electrolytes and** nutrition—Initial dehydration was treated **with intravenous fluids**. She tolerated p.o. throughout her placement and was placed on a thin pureed diet with supplemental Boost at breakfast, lunch and dinner. This was following swallow and nutrition evaluations for malnutrition. **Acid fast sputums** times three were taken for **acid fast bacilli**. On hospital day #4 sputum [**Doctor Last Name 1770**] from hospital day #2 showed positive **acid fast bacilli**. Infectious Disease consult was called. Cardiovascular—As noted above, the patient was in **rapid atrial fibrillation** initially. By hospital day #2 and throughout course the patient remained in rate control atrial fibrillation. **Chronic atrial fibrillation**. Intermittent left bundle branch block

**Table 7:** WordCloud visualization

Disorders of fluid electrolyte and acid–base balance (ICD 276)	Atrial fibrillation and flutter (ICD 427)
	

#### Error analysis

To better understand our proposed model, we performed an error analysis of the final output. There are two main types of errors: False-positive (FP) errors and True-negative (TN) errors. We list some examples to analyze these errors and find that most of the FP occur in similar ICD codes. Prediction performance heavily depends on the frequency of code occurrences in the data. This means that the higher the frequency of ICD code, the higher the AUC values. In contrast, low-frequency labels exhibit random performance. For two similar codes, if one occurs more frequently than the other, the low-frequency code may be easily predicted to be a high-frequency code, the least common 437 codes in the MIMIC-III dataset accounted for only 1% of the occurrences. For example, "007" (protozoal intestinal disease, frequency 19) was incorrectly predicted as "008" (pathogenic intestinal infection, frequency 579). The reason why the actual positive prediction was negative is that some strong positive keywords were missing, or positive indicators did not appear in the article. In cases where there are few (or no) expressions in the training set, it is extremely difficult to accurately classify real positive labels. For instance, a certain clinical note describes amoebiasis, but common discriminating keywords, such as "ameba", rarely appear in the training set. On the contrary, other disease-related words appear frequently. Our model incorrectly classifies positive instances as negative ones. In the future, we will consider better pre-processing and post-processing technologies to address the abovementioned issues.

## Conclusions

Prediction of medical codes for clinical notes is one of the pivotal tasks of biomedical NLP. The capsule network is a highly promising neural network model which has its unique advantages in biomedical text classification. In this paper, we propose the application of capsule network in the field of medical code prediction studies of clinical notes and propose the inclusion of the label embedding framework. We also compare our proposed model with various models and demonstrate that the capsule network hybrid model is indeed useful for medical code prediction. Besides, we propose a more efficient label embedding framework, which not only alleviates the problem of sparse distribution of rare medical codes but also leads to higher classification accuracy. Experimental results show that our hybrid model can effectively combine the advantages of capsule network and label embedding frameworks, and our hybrid model achieves state-of-the-art performance on the automatic medical code assignment task.

Current state-of-the-art methods in ICD code prediction are mainly based on supervised machine learning and a key challenge in the field is to reduce the model's reliance on the tag training data. As a future research direction, we would try to employ semi-supervised or migration learning in medical code prediction task and integrate biomedical knowledge to further improve the performance of our experiments.

## Data Availability

The MIMIC III datasets are available at https://mimic.physionet.org/. The pre-trained word embeddings are available at http://bio.nlplab.org/.

## Abbreviations

ICD: The International Classification of Diseases
SVM: Support vector machine
NLP: Natural language processing
BioNLP: Biomedical natural language processing
CNN: Convolutional neural network
RNN: Recurrent neural network
LSTM: Long short-term memory
Bi-LSTM: Bi-directional long short-term memory
CapsNet: Capsule network
KSI: Knowledge source integration
LRKSI: Logistic regression with knowledge source integration
RNNKSI: Recurrent neural network with knowledge source integration
CNNKSI: Convolutional neural network with knowledge source integration
CAML: Convolutional attention for multi-label classification
LEAM: Label embedding attentive model
BiCapsNet: Bi-directional Long Short-Term Memory Capsule Network
BiCapsNetLE: Bi-directional Long Short-Term Memory Capsule Network with Label Embedding
AUC: Area under the curve
TN: True-negative
FP: False-positive
